# Oral esketamine for patients with severe treatment-resistant depression: Effectiveness, safety, and tolerability of a six-week open-label treatment program

**DOI:** 10.1177/02698811251332831

**Published:** 2025-04-25

**Authors:** Jolien KE Veraart, Sanne Y Smith-Apeldoorn, Annemarie van der Meij, Jan Spijker, Robert A Schoevers, Jeanine Kamphuis

**Affiliations:** 1 Department of Psychiatry, University of Groningen, University Medical Center Groningen, Groningen, The Netherlands; 2 Department of Psychiatry, PsyQ Haaglanden, Parnassia Psychiatric Institute, The Hague, The Netherlands; 3 Depression Expertise Center, Pro Persona Mental Health Care, Nijmegen, The Netherlands; 4 Behavioural Science Institute, University of Groningen, Groningen, The Netherlands

**Keywords:** Treatment-resistant depression, oral esketamine, real-world effectiveness

## Abstract

**Background:**

Oral esketamine for patients with treatment-resistant depression (TRD) could offer certain advantages over other routes, such as intravenous or intranasal, but it has not been systematically studied in a real-world setting.

**Aims:**

Here we present results from a relatively large naturalistic study to evaluate the effectiveness, tolerability, and safety of oral esketamine in patients with TRD.

**Methods:**

One hundred eighty-five adults with severe TRD (average of 8.1 antidepressant trials plus electroconvulsive therapy in 63% without beneficial outcome) received oral esketamine treatment twice-weekly for 6 weeks with individually titrated doses ranging from 0.5 to 3 mg/kg. Outcome measures included change from baseline to week 6 on the Hamilton Depression Rating Scale (HDRS_17_), Minimal Clinically Important Difference (MCID), response, remission, self-reported symptom improvement, functioning, and side effects.

**Results:**

Oral esketamine treatment improved depressive symptom severity on the HDRS_17_ from 21.2 to 15.8 (*p* < 0.001). MCID, response, and remission rates were 47.1%, 26.8% and 15.6% respectively. In 45.9% of participants, treatment was continued after 6 weeks to maintain initial positive effects. Side effects were reported frequently but were overall well tolerated. The drop-out rate was 7.6%. We found no significant adverse effects associated with urinary tract or cognition.

**Conclusions:**

Repeated treatment with oral esketamine is effective in improving depressive symptom severity in highly treatment-resistant depressive patients. It is safe, well tolerated, and patient-friendly. Considering the level of treatment resistance, outcomes were in the range of studies investigating other routes of (es)ketamine administration.

## Introduction

Research on the antidepressant effects of ketamine and its S-enantiomer esketamine has mainly focused on intravenous (IV) and intranasal (IN) administration. IV racemic ketamine and IN esketamine have demonstrated efficacy in patients with treatment-resistant depression (TRD). Comparing outcomes across studies is hampered by the different definitions of TRD (Brown et al., 2019; Levinta et al., 2022). In most (es)ketamine studies, participants had received one or two unsuccessful antidepressant trials in the current depressive episode, complicating extrapolation to the high levels of treatment resistance and complexity in psychiatric practice (Daly et al., 2018; Diazgranados et al., 2010; Domany et al., 2019; Fava et al., 2020; Fedgchin et al., 2019; Lapidus et al., 2014; Ochs-Ross et al., 2020; Popova et al., 2019; Singh et al., 2016; Sumner et al., 2020; Zaki et al., 2023; Zarate et al., 2006). In addition, most studies have specifically excluded patients with higher levels of treatment resistance, for example, nonresponse to more than seven antidepressants or nonresponse to elektroconvulsive therapy (ECT) (Fava et al., 2020; Fedgchin et al., 2019; Hu et al., 2016; Ochs-Ross et al., 2020). Although the results of these studies are overall promising, the patient population most in need would often be excluded and likely has an even more untoward prognosis (Veraart et al., 2018). Studies in patients with a depression that is refractory to multiple antidepressants and ECT are limited, although positive effects of IV and oral (es)ketamine have been observed (Rodrigues et al., 2022; Thomas et al., 2018; Vendrell-Serres et al., 2024; Veraart et al., 2021a).

In addition to the variation in treatment resistance, (es)ketamine studies have applied relatively restrictive criteria regarding comorbidities such as psychotic symptoms or suicidality (Daly et al., 2018; Domany et al., 2019; Fava et al., 2020; Fedgchin et al., 2019; Lapidus et al., 2014; Ochs-Ross et al., 2020; Popova et al., 2019; Singh et al., 2016; Zarate et al., 2006). This may be overly cautious (Veraart et al., 2021b) and limits the generalizability of findings to regular clinical practice, where comorbidities are the rule rather than the exception (Otte, 2008). Larger studies with samples closely mirroring the level of treatment resistance and diversity of patients encountered in day-to-day (tertiary) clinical practice are therefore warranted.

Both the IV and IN routes of administration need application under direct medical supervision, which may hamper clinical scalability and increase costs (Ross and Soeteman, 2020). Although intramuscular (IM) and subcutaneous (SC) administration also appears to be effective (Cigognini, 2022; Cavenaghi, 2021; George et al., 2017; Lee, 2023; Loo et al., 2016, 2023), they may cause local irritation or pain (Andrade, 2017). Oral administration of generic (es)ketamine could be an interesting option that is less invasive, patient-friendly and potentially less costly than other variants (Andrade, 2022). Moreover, it provides a practical strategy for long-term use at home, as repeated (es)ketamine dosing is increasingly being recognized as an important strategy to maintain initial antidepressant effects (Daly et al., 2019; Smith-Apeldoorn et al., 2022).

Following oral administration, the first-pass effect leads to a bioavailability of 8%–24%, which requires an adjustment in the dose (Fanta et al., 2015; Peltoniemi et al., 2012). Although oral (es)ketamine administration has not been compared to IV and IN administration directly, positive results with oral ketamine for depression have been found in small, uncontrolled studies (Glue et al., 2020; Rosenblat et al., 2019; Schoevers et al., 2016). In addition, significant changes in depressive symptoms were observed after 2–6 weeks of oral ketamine treatment when compared to placebo in three randomized controlled trials (RCTs) (Arabzadeh et al., 2018; Domany et al., 2019; Jafarinia et al., 2016).

In the Netherlands, esketamine is the registered generic formulation for anesthesia and would thus be the likely candidate for oral application. Although esketamine is a more potent N-methyl-D-aspartate (NMDA) receptor antagonist and the antidepressant efficacy of IN esketamine has been demonstrated (Daly et al., 2018; Ochs-Ross et al., 2020; Popova et al., 2019), preclinical research suggests that arketamine may show more potent antidepressant effects and fewer side effects (Chang, 2019; Yang, 2018; Zhang, 2014). Nevertheless, inconclusive results are reported for clinical studies with 0.5 mg/kg IV arketamine. A small open-label pilot (*n* = 7) (Leal, 2021) found significant antidepressant effects whereas two RCTs (*n* = 10 and 102) reported a lack of antidepressant effects of arketamine (Atai Life Sciences, 2023; Leal, 2023).

To date, small case series, including a pilot study from our group, are suggestive of effects of oral esketamine comparable to oral racemic ketamine (Paslakis et al., 2010; Smith-Apeldoorn et al., 2021). In a randomized placebo-controlled trial, a fixed low dose of 30 mg three times daily for 6 weeks was compared to placebo in 111 participants (Smith-Apeldoorn et al., 2024). The treatment showed no benefit on the HDRS_17_ total score (*p* = 0.626), which was likely attributable to the dosing regimen. The aim of this article is to provide data on the clinical effectiveness and safety of an open-label, individually titrated higher-dose oral esketamine treatment for a relatively large sample of patients with TRD. Treatment was offered according to an off-label protocol in three specialized depression treatment centers in the Netherlands.

## Methods

### Participants and study design

We conducted this multicenter, 6-week open-label study to assess the effectiveness, safety and tolerability of twice-weekly oral esketamine. The participants were treated according to an off-label “compassionate use” protocol that was evaluated by the Medical Ethics Committee of the University Medical Center Groningen as not subject to the Dutch law on medical research involving human subjects (file number 2017/446). Oral esketamine treatment was offered to inpatients and as daycare to outpatients at the University Medical Center in Groningen, Pro Persona Mental Health Care in Nijmegen, and PsyQ Parnassia Psychiatric Institute in The Hague. All participants gave written informed consent for off-label treatment and collection, analysis and publication of anonymized data.

The participants in this study came from two sources. Seventy-seven patients with TRD had previously participated in our double-blind RCT comparing thrice daily 30 mg oral esketamine versus placebo for 6 weeks, followed by a follow-up wash-out phase of 4 weeks(Smith-Apeldoorn et al., 2024). Patients were offered off-label treatment following their involvement in the RCT to ensure that they had the opportunity to try esketamine treatment, even if they had previously been in the placebo group. The treatment was offered after the 4-week follow-up phase of the RCT, prior to the unblinding of the group allocation. We adopted this strategy in close collaboration with patient experts because we deemed it unethical to enroll participants in the RCT without offering all patients access to a potentially effective intervention. To be eligible for the RCT, patients had to meet the following requirements: unipolar depressive disorder, moderate-to-severe depression (HDRS total score > 18), and failure of adequate trials with at least three different classes of antidepressant medication lifetime (Smith-Apeldoorn et al., 2019). The primary outcome for these patients (depressive symptom severity and (partial) response) was also briefly reported in a separate paper primarily focusing on the results of the RCT (Smith-Apeldoorn et al., 2024).

The current study provides a more detailed description of the population and, apart from the primary outcome, also reports adverse events, self-reported depressive symptom severity, and functioning. Moreover, the current study also reports the outcomes of a much larger sample of 108 patients who did not participate in the RCT, significantly expanding the sample previously discussed. These participants had not participated in the RCT, for instance, because they suffered from psychotic symptoms or were referred after the inclusion period had ended (February 2021).

Oral esketamine was prescribed off-label, based on the fact that they had unsuccessfully tried a range of conventional treatment steps according to the Netherlands guidelines for the treatment of depression (Spijker et al., 2013). The results of oral esketamine treatment for patients with treatment-resistant bipolar depression will be reported in a separate article.

Exclusion criteria included the inability to provide informed consent, active substance use or alcohol use disorder, somatic contraindications (i.e. intolerability or allergy to (es)ketamine, severe cardiac and vascular disease, severe liver or kidney function impairment, increased intracranial pressure, poorly controlled hypertension, pregnancy or lactation). If patients had other conditions that could increase the risk of negative outcomes, extra caution was applied, for instance, with more intensive monitoring of symptoms and possible side effects. These conditions included a history of or current psychotic symptoms and a history of substance use or alcohol use disorder. Patients with suicidal ideations were not excluded.

### Treatment protocol

The patients received a total of 12 doses of oral esketamine in a liquid formulation over the course of 6 weeks. Subjects continued their regular antidepressant and other psychiatric medications. We advised them to taper down the use of benzodiazepines and *Z*-drugs to a maximum of 2 mg/day before esketamine treatment initiation. We did not use a fixed protocol for the tapering of benzodiazepines prior to the start of the esketamine treatment, it was individualized for each patient. In some cases, the tapering process might have been completed shortly before the initiation of esketamine treatment, but overall the benzodiazepine dosages were relatively low, and patients were not having withdrawal reactions when they started esketamine.

Esketamine doses started at 0.5 or 1 mg/kg and were increased in increments of 0.5 mg/kg to a maximum of 2.0 mg/kg (June 2017–April 2019) or 3.0 mg/kg (May 2019–June 2023). The starting dose and maximum dose in our protocol were increased based on publications that were helpful to gain a better understanding of the interindividual variations in oral esketamine bioavailability (Andrade, 2019) in combination with our own increasing clinical experience; we found that many patients experienced antidepressant effects only after several dose escalations. Still, we kept a personalized dosing strategy; if patients were particularly anxious about the acute effects, clinicians sometimes opted for the lower starting dose also after April 2019. The dose titration was guided by shared decision-making between the patient and the clinician, taking into account the acute effects, tolerability, and antidepressant response.

Patients were under the care of a nurse during 2–3 h in the clinic. Blood pressure and heart rate were measured at baseline and 30 and 90 min after esketamine ingestion. Intolerable acute side effects were followed by a reduction of the dose. Paracetamol, ondansetron, or antihypertensive medication were used to manage side effects such as headaches, nausea, and hypertension if indicated.

After 6 weeks, an elaborate evaluation was planned with the patient, their close ones, their referring psychiatrist, and the clinician providing esketamine treatment. In case of a subjective and/or measured improvement in depressive symptoms or quality of life, this was weighed against the possible risks of longer-term esketamine administration, to come to an informed decision on starting maintenance treatment. Other relevant considerations for this decision involved the (lack of) alternative treatment options, and the (lack of) reimbursement by health insurance providers, which needs to be requested separately for each patient. In the maintenance phase, patients continued with outpatient “at-home” oral esketamine treatment.

### Outcome measures

Questionnaires on outcome measures were assessed over the course of the 6 weeks, with the last measurement prior to the last dosing session in week 6. The level of treatment resistance was assessed with the Dutch Measure for Quantification of Treatment Resistance in Depression (DM-TRD), evaluating information about clinical conditions, psychiatric comorbidity, and failed treatments for the current episode. The DM-TRD contains 11 items and scores range from 2 to 27 (higher scores indicating worse treatment outcome; Peeters et al., 2016).

The primary outcomes were depressive symptom severity change from baseline to week 6 measured by the Hamilton Depression Rating Scale (HDRS_17_), with higher scores indicating greater severity of depressive symptoms (Hamilton, 1967), response (⩾50% reduction in HDRS_17_ score), remission (HDRS_17_ score ⩽ 7) and the minimal clinically important difference (MCID). The MCID is defined as “the minimum change in a measurable outcome in which the patient perceives a difference because of an intervention” (Masson and Tejani, 2013). Recognition of the use of MCID values for clinical trials and for the decision-making process in clinical practice is increasing (Hudgens et al., 2021; Masson and Tejani, 2013; McIntyre et al., 2021; Turkoz et al., 2021). A study with 681 patients with nonpsychotic MDD yielded an MCID of 27.1% decrease in depressive symptom severity measured by the HDRS_17_ (Rush et al., 2003).

In addition, we report the percentage of patients suffering from suicidality (an HDRS_17_ suicidal ideation item score of >0 at baseline) who achieve a decrease in the severity of suicidality. We analyzed the self-reported depressive symptoms, assessed by the Inventory of Depressive Symptomatology – Self Rated (IDS-SR, with higher scores indicating greater severity of depressive symptoms (Rush et al., 1986)), prior to treatment session 1 (week 1), session 3 (week 2), session 5 (week 3), session 7 (week 4), session 9 (week 5) and session 11 (week 6). Functioning was assessed with the World Health Organization Disability Assessment Schedule II (WHODAS II, (Epping-Jordan and Ustun, 2000)), a 36-item assessment of health and disability with higher scores indicating greater levels of disability. If a participant does not have work or study, the number of questions is reduced to 32.

A possible dose-response relationship was investigated by comparing the mean esketamine dose in week 6 between the groups of patients that did and did not reach MCID.

### Adverse event monitoring

The incidence of side effects was calculated by comparing the Systematic Assessment for Treatment Emergent Events (SAFTEE; Levine and Schooler, 1986) at baseline and in week 6. The SAFTEE assesses the side effects of any biologic treatment through a systematic review of organ systems. An increase of symptoms from “not present” to “moderate” or “mild” to “severe” was defined as moderate discomfort, and an increase of “not present” to “severe” as severe discomfort. Urinary tract symptoms were assessed using the Interstitial Cystitis Symptom Index and Problem Index (ICSI-ICPI; O’Leary et al., 1997) at baseline and after 6 weeks. The ICSI contains four items regarding urination frequency, nocturnal urination, sudden need for urination, and pelvic pain complaints. The maximum score is 20, scores < 12 indicate mild interstitial cystitis and ⩾12 indicate severe interstitial cystitis. The ICPI assesses to what extent the symptoms create problems in daily life, with a maximum score of 16. Cognitive functioning was assessed at baseline and after 6 weeks with the Montreal Cognitive Assessment (MoCA; Nasreddine et al., 2005), a screening tool for cognitive impairment with a maximum score of 30 and higher scores indicating less impairment. As doses were individually titrated based on antidepressant effects and tolerability, we assumed that the final dose in week 6 was the one most clinically effective. Therefore, we analyzed dissociative symptoms before and 30 min following esketamine administration in week 6 with the Dissociation Tension Scale (Dissoziations-Spannungs-Skala (DSS); Stiglmayr et al., 2010), designed to record short-term shifts in dissociative symptoms. The DSS contains 21 items that are scored on a scale from 0% to 100%.

### Statistical analyses

Results of descriptive statistics of demographic and clinical characteristics are reported. To compare scores of baseline and week 6, a paired-sample *t*-test was performed for the continuous variables and a Wilcoxon signed-rank test with the nominal data. MCID, response, and remission rates are reported for both the full sample and a complete-case subset. We performed no imputation to handle missing data. The IDS-SR data were analyzed using a linear mixed model, with a fixed effect for measure (weekly) and a random effect for patients. SAFTEE scores from baseline and week 6 were compared, and frequency scores of a moderate or severe increase or onset for each reported side effect were calculated. The change in DSS total scores before and 30 min after administration of the last dose was calculated. Statistical analyses were conducted using SPSS, and tests were considered to be significant at *p* < 0.05.

## Results

### Baseline characteristics

The 185 included patients were treated with oral esketamine from April 2017 to June 2023. Table 1 shows baseline demographic and clinical characteristics. The mean duration of the current depressive episode was more than 6 years. More than two-thirds of the patients suffered from psychiatric comorbidities (67.1%), including personality disorder (38.2%), anxiety disorder (24.9%), and posttraumatic stress disorder (PTSD, 13.9%). A total of 43.6% had attempted suicide at least once in their lifetime. On average, patients had tried 8.1 antidepressants lifetime, and 63.0% had tried ECT. The mean DM-TRD total score was 19.6 (SD 3.3); more details on the level of treatment resistance with individual DM-TRD item scores can be found in the Supplemental Information.

**Table 1.** Baseline and clinical characteristics.

Patient characteristics (n*)	Total sample (*n* = 185)	
	*N*	%
Sex (185)		
Male	62	33.5
Marital status (147)		
Single	46	24.9
Married/living together	87	47.0
Divorced	11	5.9
Widow(er)	3	1.6
Comorbid psychiatric disorder (173)	116	67.1
Personality disorder	66	38.2
Anxiety disorder	43	24.9
PTSD	24	13.9
OCD	7	4.0
ASS	15	8.7
Eating disorder	10	5.8
ADHD	13	7.5
Substance use disorder	4	2.3
Schizophrenia spectrum and other psychotic disorders	3	1.7
Other psychiatric disorder^	3	1.7
Current use of AD medication (173)		
SSRI	45	26.0
SNRI	22	12.7
Lithium/AP/T3/stimulant	62	35.8
TCA	54	31.2
MAOI	21	12.1
Other antidepressant medication^#^	73	42.2
Current use of BZD (173)		
None or ⩽2 mg lorazepam equivalent/day	121	69.9
>2 mg lorazepam equivalent/day	52	30.1
History of ECT (173)	109	63.0
Suicide attempt lifetime (172)	75	43.6
Current PT treatment (173)	84	48.6
	Mean	SD
Age in years (185)	52.9	14.1
Duration of depression in months (185)	75.6	84.2
No. of depressive episodes lifetime (184)	8.2	18.0
Age of onset 1st depr. episode (184)	28.6	15.2
No. of prior AD trials lifetime (171)	8.1	3.5
No. of psychotherapies lifetime (165)	3.7	2.7
Baseline HDRS_17_ score (138)	21.2	6.0
Baseline IDS-SR score (145)	44.9	11.5
Baseline DM-TRD score (126)	19.6	3.3

AD: antidepressant; ADHD: attention-deficit/hyperactivity disorder; AP: antipsychotic medication; ASS: autism spectrum disorder; BZD: benzodiazepines; DM-TRD: Dutch measure for quantification of treatment resistance in depression; ECT: electroconvulsive therapy; HDRS: Hamilton depression rating scale; IDS-SR: inventory of depressive symptomatology – self-report; OCD: obsessive-compulsive disorder; PT: psychotherapeutic; PTSD: posttraumatic stress disorder; SNRI: selective serotonin and norepinephrine reuptake inhibitor; SSRI: selective serotonin reuptake inhibitor; T3: triiodothyronine; TCA: tricyclic antidepressant; MAOI: monoamine oxidase inhibitor.

* Number of patients for whom data was available.

^ Other psychiatric disorders: somatic symptom disorder, intellectual disability, depressive disorder due to another medical condition.

# Other antidepressant medication: mirtazapine, bupropion, agomelatine, vortioxetine, mianserin, etc.

### Drop-out

The overall attrition rate was 7.6% (*n* = 14). Table 2 shows the reasons for discontinuation and the number of sessions patients received before withdrawal. Reasons included side effects in eight patients and lack of improvement in eight patients as well.

**Table 2.** Drop-out.

Patient	No. of sessions	Reason for discontinuation
1	2	Electric shock sensations in the head
2	2	Lack of improvement
3	3	Challenging experience, dizziness, nausea
4	3	Lack of improvement and (unknown) side effects
5	4	Increase in PTSD-related symptoms
6	5	Lack of improvement, nausea, dissociation
7	5	Lack of improvement
8	5	Travel time
9	6	Anxiety, increase in PTSD-related symptoms
10	6	Covid restrictions
11	9	Lack of improvement
12	9	Lack of improvement
13	9	Lack of improvement, anxiety
14	10	Lack of improvement, challenging experience, nausea, vomiting, urinary incontinence

Number of sessions before discontinuation and reasons for drop-out during the esketamine treatment.

PTSD: posttraumatic stress disorder.

### Depression outcomes

HDRS_17_ total score data were missing at baseline and week 6 in 12 patients, only at baseline for another 3 patients, and at week 6 for another 18 patients. As 14 patients had ended treatment before the endpoint, HDRS_17_ data were available at both time points in 138 patients. The patients without an HDRS_17_ total score in week 6 (due to drop-out or missing data) did not differ significantly from the other patients at baseline in terms of depression severity (HDRS_17_ total score) or treatment resistance (DM-TRD total score).

The mean HDRS_17_ total score decreased from 21.2 (SD 6.0) to 15.8 (SD 7.5) points (mean decrease 25.7%, SD 29.0, range of change in HDRS_17_ total score −40.0% to 86.4%). We found a significant decrease of depressive symptom severity: *t*(137) = 10.56, *p* < 0.001.

Among patients with HDRS_17_ data available at baseline and week 6, 47.1 % (*n* = 65) reached the MCID (35.1% of the ITT population). Response was observed in 26.8% (*n* = 37, 20.0% of the ITT population) and remission in 15.6% (*n* = 22, ITT 11.9%).

Among the 114 patients with an HDRS_17_ suicidal ideation item score of >0 at baseline, 36 (31.6%) achieved complete absence of suicidal ideation (score = 0 at week 6). In 63 patients (55.3%), the suicidality score decreased by at least 1 level (e.g. from 2 to 1 or from 2 to 0).

**Figure 1.**
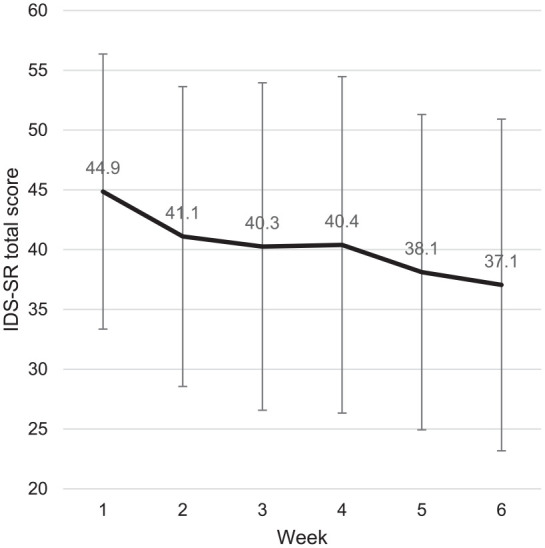
Weekly mean IDS-SR total score and standard deviation.

The amount of IDS-SR data available and the mean total scores per timepoint are shown in Table 3. IDS-SR scores showed a significant decrease after 6 weeks compared with baseline (β = −8.08, 95% CI: −9.91 to −6.26, *p* < 0.001). The IDS-SR mean total score at every other timepoint also showed a significant decrease from baseline (Table 3). When compared to the previous timepoint, a significant difference was found from weeks 1 to 2 and weeks 4 to 5, although the mean total scores continued to decrease over the course of 6 weeks (Figure 1).

**Table 3.** Weekly IDS-SR scores.

Measure	Week 1	Week 2	Week 3	Week 4	Week 5	Week 6
*N*	145	123	127	102	99	132
Mean (SD)	44.9 (11.5)	41.1 (12.5)	40.3 (13.7)	40.4 (14.1)	38.1 (13.2)	37.1 (13.9)
Coefficient (*p*)	n.a.	−8.1 (<0.001)*	−6.9 (<0.001)*	−5.2 (<0.001)*	−4.6 (<0.001)*	−3.6 (<0.001)*
Contrast estimate (p)	n.a.	−3.6 (<0.001)*	−1.0 (0.096)	−0.5 (0.422)	−1.7 (0.016)*	−1.2 (0.071)

Amount of IDS-SR data per timepoint, mean and standard deviation of IDS-SR total scores, coefficient with target set to week 1, contrast estimate versus previous timepoint.

* Statistical significance at *p* < 0.05.

n.a.: not applicable,

Eighty-five patients (comprising 45.9% of the total sample and 49.7% of patients who completed the 6-week treatment) continued maintenance treatment after 6 weeks. Also, among the 44 patients with missing endpoint HDRS_17_ data, 10 patients (22.7%) chose maintenance treatment, suggesting subjectively experienced positive effects of treatment. Of patients for whom HDRS_17_ data was available, 73 did not reach MCID. Out of these, 28 patients (38.4%) expressed clinically relevant positive effects and continued treatment. The highest proportion of patients who wished to continue treatment after 6 weeks was among those who reached MCID; out of a total of 65, 46 patients (70.8%) continued.

### Functioning

The mean total WHODAS II score excluding the work section (data available from 55 patients) decreased from 50.2 (SD 16.4) to 41.5 (SD 17.0), with a mean change of 17.4% (*p* < 0.001).

Mean pre- and post-treatment WHODAS II scores per domain are shown for MCID and non-MCID groups in Table 4. A significant decrease was found for the WHODAS II sum score in both groups and for all individual domains in the MCID group. Although the mean change was consistently higher in the MCID group, the differences between the groups were not statistically significant. For two patients, information on MCID was missing.

**Table 4.** WHODAS II scores.

WHODAS II Domains	MCID (*n* = 24)				non-MCID (*n* = 29)				Comparison of mean change between groups, *p*
	Mean (SD) pre-treatment	Mean (SD) post-treatment	Mean (SD) change	*p*	Mean (SD) pre-treatment	Mean (SD) post-treatment	Mean (SD) change	*p*	
Total (*n* = 53)	47.4 (16.0)	35.5 (14.5)	12.0 (14.5)	0.001*	52.0 (16.3)	45.1 (16.8)	6.9 (12.0)	0.003*	0.168
Cognition (*n* = 53)	48.8 (22.0)	38.1 (22.5)	10.6 (21.2)	0.045*	54.8 (22.6)	47.1 (22.1)	7.8 (16.7)	0.030*	0.584
Mobility (*n* = 53)	34.4 (23.9)	22.1 (19.8)	12.2 (23.4)	0.016*	34.5 (27.6)	28.0 (25.8)	6.5 (18.3)	0.054	0.318
Self-care (*n* = 53)	29.2 (23.4)	16.7 (19.3)	12.5 (18.5)	0.002*	28.3 (25.9)	21.7 (18.7)	6.5 (22.1)	0.245	0.299
Getting along (*n* = 53)	56.9 (25.7)	45.5 (21.1)	11.5 (22.5)	0.009*	64.7 (23.2)	56.5 (29.2)	7.1 (26.0)	0.159	0.530
Life activities (*n* = 53)	62.9 (26.1)	42.9 (17.3)	20.0 (22.8)	<0.001*	61.0 (28.3)	49.3 (33.8)	11.7 (32.1)	0.057	0.294
Participation (*n* = 53)	51.4 (17.0)	41.8 (19.8)	9.5 (18.4)	0.024*	61.1 (19.8)	56.8 (18.1)	4.3 (15.7)	0.245	0.269

Distribution of WHODAS II scores pre- and post-treatment; change in WHODAS II sum score and component scores between pre- and post-treatment using Wilcoxon Signed Ranks Test; comparison of WHODAS II scores change between MCID and non-MCID groups with independent samples *t*-test.

* Statistical significance at *p* < 0.05.

MCID: minimal clinically important difference; WHODAS: World Health Organization disability assessment schedule.

### Dose-response relationship

We found no significant difference in the mean final esketamine dose in week 6 between the groups of patients that did and did not reach MCID. The mean final dose was 2.1 mg/kg (SD 0.7, range: 0.25–3.5 mg/kg) versus 2.2 mg/kg (SD 0.8, range: 0.5–3.0 mg/kg) respectively, *t*(99) = 0.839, *p* = 0.404. In the group that did reach MCID, there was one patient receiving 3.5 mg/kg in the end, as no acute or antidepressant effects occurred on lower dosages. This was done per clinical judgment and discussed in the esketamine treatment separately, as no other treatment options were available.

### Safety and tolerability

SAFTEE data at baseline and week 6 were available for a total of 126 patients. A *moderate* increase of symptoms was reported 70 times and included weight loss, trouble sleeping, constipation, headache, feeling nervous or hyper, dry mouth, muscle cramps or stiffness, frequent need to urinate, strange taste in mouth, diminished mental and unable to sit still. A *severe* increase in symptoms was reported 24 times; this primarily concerned sexual dysfunction: delayed or absent orgasm in eight patients, problems with sexual arousal in five patients, loss of sexual interest in two patients and menstrual irregularities in one patient. Headache, apathy/emotional indifference, poor memory, clenching of teeth at night, hot flashes, and blurred vision were reported less often. A detailed overview of increases in SAFTEE items can be found in the Supplemental Information.

The mean MoCA total score improved significantly after treatment. Baseline and week 6 MoCA data were available for 71 patients, with mean scores of 25.6 (SD 3.4) and 26.8 (SD 2.8) respectively. An increase of MoCA total score was found in 44 (62.0%) patients and ranged from 1 (*n* = 12) to 8 (*n* = 2) points. A decrease was observed in 19 (26.8%) patients and ranged from 1 (*n* = 7) to 4 (*n* = 1) points.

There was no significant effect of esketamine on the ICSI and ICPI total score in 82 patients with baseline and week 6 data. Mean total scores decreased from 2.2 (SD 3.0) to 1.9 (SD 2.6) for the ICPI and the ICSI remained stable at 3.1 (SD 2.9 at baseline and SD 2.6 at week 6). An increase in symptoms on the ICSI was found for 27 patients (32.9%) and ranged from 1 (*n* = 11) to 5 (*n* = 2) points. An increase in ICPI scores was reported in 21 (25.6%) patients and ranged from 1 (*n* = 13) to 3 (*n* = 3) points.

Comparing the DSS scores before the administration of esketamine and after 30 min in week 6 (in 71 patients with data available), the mean total score increased with 297.6 points (SD 419.6).

An increase in dissociative symptoms was reported by 77.5% of patients (*n* = 55).

We performed no formal assessment of (es)ketamine craving; however, no (es)ketamine-seeking behavior was observed, nor did any patient spontaneously report craving.

## Discussion

This open-label study of repeated oral esketamine treatment in 185 patients with high TRD is substantially larger than earlier investigations with oral (es)ketamine, describing patient groups ranging between 1 and 80 participants (Meshkat et al., 2023). We found that twice-weekly dosing for 6 weeks resulted in a significant improvement in depressive symptom severity on the HDRS_17_ and IDS-SR, with treatment being well tolerated.

Comparing our results with those of other esketamine trials in TRD patients, the response and remission rates in our sample (26.8% and 15.6%, respectively) were lower than those reported in two studies on IN esketamine (Fedgchin et al., 2019; Popova et al., 2019) and similar to the results in one study (Ochs-Ross et al., 2020). In the first two trials, response rates were 54.1% (56 mg), 53.1% (84 mg) and 69.3% (56 or 84 mg) and remission rates were 36.0% (56 mg), 38.8% (84 mg) and 52.5% (56 or 84 mg). However, differences between the outcomes of these IN esketamine RCTs and our off-label program should be interpreted with great caution, as our sample was substantially more treatment resistant. In the three IN esketamine RCTs (Fedgchin et al., 2019; Ochs-Ross et al., 2020; Popova et al., 2019), the proportion of patients who had failed more than two adequate antidepressant trials in the current depressive episode ranged between 31.6% and 39.7%, whereas in our sample this proportion was 83.9%, with a mean DM-TRD score of 19.6, on average 8.1 failed antidepressant trials and unsuccessful ECT in 63% (DM-TRD item 7 in Supplemental material). The relevance of these differences is illustrated by the fact that even the response and remission rates in the control groups of these RCTs were higher than those found in our sample (response in 38.9% and 52.0%, remission in 30.6%, and 31.0%). As expected, patients with higher levels of treatment resistance have poorer treatment outcomes in both regular (Fekadu et al., 2009a, 2009b; Trivedi et al., 2006) and (es)ketamine treatments (Levinta et al., 2022). However, when comparing our results with the landmark Sequenced Treatment Alternatives to Relieve Depression (STAR*D) study (Rush et al., 2006), the response and remission rates we observed exceeded those found in STAR*D (16.3% and 13.0%) in patients who had “only” four unsuccessful treatment steps with regular antidepressants and/or cognitive behavioral therapy. On top of that, the median length of the current depressive episode in the STAR*D study was 7.8 months, compared to 75.6 months in our sample, with longer index episodes generally predicting lower remission rates (Sinyor et al., 2010).

The difference in the response and remission rates found in the studies by Fedgchin et al. (2019) and Popova et al. (2019) could additionally be explained by the discrepancy between rigorously controlled efficacy studies and real-world effectiveness in clinical practice (Alnefeesi et al., 2022). This pattern was also shown in off-label IV treatment with racemic ketamine that resulted in underwhelming response (18%–44%) and remission (13%–16%) rates (McIntyre et al., 2020b; Sakurai et al., 2020; Thomas et al., 2018; Vestring et al., 2024) in comparison to RCTs. The differences can be attributed to differing levels of treatment resistance but also to the exclusion of patients with additional clinical characteristics such as bipolar disorder, psychotic symptoms or suicidality in RCTs.

In more than half of the patients with suicidality (55.3%), the score on the suicidal ideation item of the HDRS_17_ decreased by at least one level, and almost a third of the patients (31.6%) achieved complete remission of suicidality. These results are in line with the response and remission rates of 44% and 27%, respectively, observed in pooled data from five RCTs (*n* = 128) with IV racemic ketamine (Ballard et al., 2018). Meta-analyses, including RCTs investigating the effect of IV racemic ketamine compared to saline or midazolam on suicidal ideation, report a significant reduction within the first 72 h (Jollant, 2023). On the other hand, in four large RCTs, IN esketamine did not differ from placebo (Jollant, 2023). It should be noted that in RCTs investigating suicidal ideation, significant effects are also observed in control groups, particularly in inpatients. Empathetic support and connection can impact suicidal ideation in the short term and may also have played a role in the observed effects on suicidality in our population. Furthermore, the effects on suicidal ideation could also be explained by (es)ketamine’s antidepressant effects. Some studies suggest that the effects may be partly independent (Ballard, 2014, 2018; Wilkinson et al., 2018), though not all research supports this (Apeldoorn, 2017; Murrough, 2015; Price, 2014).

Despite the gradual dose titration schedule, and literature suggesting a later onset of antidepressant effects with oral administration when compared to IV or IN administration (McIntyre et al., 2020a), we observed antidepressant effectiveness based on IDS-SR scores after two treatment sessions. Nevertheless, the benefits became more pronounced over the course of 6 weeks, implying that there is merit in extending the duration of treatment beyond these 6 weeks. The benefit of prolonged treatment has also been demonstrated in the study by Reif et al. (2023), reporting increased remission rates after 32 weeks of IN esketamine treatment when compared to the end of the acute treatment phase.

In this sample, 45.9% of patients benefited from the treatment and decided, together with their clinician, to continue maintenance treatment. This proportion corresponds to percentages found in previous studies (31%–50 %) (Archer et al., 2018; Daly et al., 2019; Sakurai et al., 2020; Wilkinson et al., 2018). Not all patients who wished to continue treatment after the induction phase had reached HDRS_17_ response or remission thresholds. We observed that even partial symptom improvement could have significant meaning for patients in the clinical setting. Prior research has demonstrated that patients consider quality of functioning and positive affect to be important in relief from depression (Demyttenaere et al., 2015a, 2015b). As observed in the WHODAS II outcomes, oral esketamine treatment improved functioning in all domains: cognition, mobility, self-care, getting along, life activities, and participation. Previous trials investigating the antidepressant effects of IV ketamine and IN esketamine also found improvements in functioning (McIntyre et al., 2020b; Popova et al., 2019; Wajs et al., 2020; Zhou et al., 2022). Even when response is not detected on depression-specific instruments such as the HDRS_17_, patients might experience an improvement in functioning or overall wellbeing. In patients with very hard-to-treat depression, where treatment protocols are exhausted and offer no alternatives, such improvements are relevant and worthwhile for both patients and carers. Using a 50% decrease in depression symptom severity may underestimate this improvement (Zimmerman and Lin, 2023).

RCTs studying oral racemic ketamine in MDD indicated good safety and tolerability, with no or only mild dissociative symptoms and no signs of abuse or dependence (Arabzadeh et al., 2018; Domany et al., 2019; Jafarinia et al., 2016). In our study, only 8 of 185 patients (4.5%) dropped out because of side effects. The types and frequencies of reported side effects were, in general, similar to those reported with oral (es)ketamine treatment elsewhere. No clinically relevant symptoms of urinary tract problems, cognitive impairment, or craving were observed in our study. The noninvasive oral route of administration was acceptable for patients, and they felt confident to change to maintenance treatment at home after the first phase of clinical treatment. However, as the therapeutic potential of (es)ketamine maintenance treatment is becoming increasingly evident (Smith-Apeldoorn et al., 2022), implementing monitoring programs with a thorough investigation of the risk of abuse and long-term side effects is warranted.

Of note, a moderate or severe increase in sexual dysfunction symptoms was most often reported as a side effect by 13.5% of participants (*n* = 17/126). In abusers of street ketamine, sexual dysfunction such as female sexual dysfunction and erectile dysfunction has been described (Jang et al., 2012; Jong, 2023; Suppiah et al., 2016; Yang et al., 2018). Mechanisms underlying (es)ketamine-related sexual dysfunction remain poorly understood. In rats, long-term ketamine administration caused decreased erectile responses, loss of smooth muscle content in the corpus cavernosum, and corporal apoptosis (Shang, 2017). It would be interesting to explore how the risk of sexual side effects may differ across various routes of administration, considering their distinct pharmacokinetic profiles. The extensive first-pass metabolism after oral administration influences systemic exposure to metabolites of (es)ketamine. Unfortunately, assessment of sexual dysfunction is uncommon in other studies with (es)ketamine for depression (Short et al., 2018; Williamson et al., 2022). Given our findings, we would argue for routine, standardized assessment of sexual functioning in (es)ketamine studies, especially in the case of long-term treatment, as relying on the spontaneous report of patients leads to underestimation of such symptoms (Clayton et al., 2014).

Regarding tolerability and safety, the oral route of (es)ketamine administration is the least invasive and has been associated with fewer acute side effects than IV or IN routes, potentially rendering it more suitable for at-home maintenance treatment after a first phase of clinical treatment and dose titration (Irwin et al., 2013; Schoevers et al., 2016). Although at-home treatment needs to be monitored well, the requirement of clinical application and supervision for IN and IV administration rapidly leads to saturation of (es)ketamine treatment programs and consequently limited access. If efficacy is comparable, implementing at-home treatment with oral (es)ketamine could mitigate scalability issues related to treatment capacity.

### Limitations

Major limitations of this study are the lack of blinding and a control group to compare the effects of the intervention and the duration of follow-up. The open-label design of this study can result in an overestimation of the effects of oral esketamine. We believe expectancy bias might have influenced the results. The patients often considered this off-label treatment as a “last resort” and may have read about the promising results of this novel treatment option (Gallagher et al., 2023). Nevertheless, a larger number of previously failed interventions is associated with a smaller placebo effect in patients with TRD (Brunoni et al., 2009; Jones et al., 2021; Loo et al., 2023; Razza et al., 2018), which would suggest that the patients in our sample were not very susceptible to placebo effects.

Worth mentioning is that our study sample consisted partly of prior RCT participants (42% of the total study sample). It cannot be excluded that the experienced effect in the RCT (attributed either to expectancy to be in the esketamine or in the placebo group) influenced their choice to take part in the off-label esketamine treatment. However, we expect this affected our findings to a very low or negligible extent, as both in the esketamine and in the placebo group, 65% of patients entered the off-label treatment program (Smith-Apeldoorn et al., 2024).

Controlled trials directly comparing (es)ketamine with evidence-based antidepressant treatments are warranted. A randomized clinical trial directly comparing the efficacy of oral esketamine treatment versus ECT is currently being conducted by our team. Furthermore, a longer study duration would allow for the identification of effects that occur only after long-term use of esketamine, such as the development of tolerance, urinary tract symptoms, sexual dysfunction or cognitive side effects. We will analyze the results of maintenance esketamine treatment in our treatment centers in a follow-up study.

We encountered missing data, which unfortunately more easily occurs in real-world studies without additional funding for rigorous data collection. Several patients found the amount of questionnaires burdensome. To reduce the burden of questionnaires for patients and therapists in future ketamine studies while maintaining comprehensive monitoring of potential side effects, we advise using the Ketamine Side Effect Tool (Bayes et al., 2022). This questionnaire is specifically designed for (es)ketamine treatment and allows for monitoring of relevant adverse events that may arise. To manage the missing data limitation, we reported MCID, response, and remission rates for both the per-protocol and the intention-to-treat population of patients with a baseline HDRS_17_ score available.

A significant limitation of this study is that we did not compare the clinically observed effects with plasma concentrations of esketamine and its metabolites. Oral esketamine shows a high first-pass effect and interpersonal variability (Fanta et al., 2015). Reference values for esketamine and its metabolites are not yet available for antidepressant treatment. We performed individual titration by monitoring clinical effects and tolerability after each administration, which appeared useful. The titration strategy has shown clinical utility in other (es)ketamine studies (George et al., 2017; Lai et al., 2014; Loo et al., 2016, 2023; Popova et al., 2019; Vestring et al., 2024) and is customary for other types of medication such as antidepressants, antipsychotics, and stimulants (Caffrey and Borrelli, 2020). We did not identify a dose-response relationship; however, this analysis may be biased because dose-escalation was specifically applied to patients who did not respond to treatment. Other factors that could contribute to the lack of a clear dose-response relationship were not taken into account in this study. These factors include substantial interindividual differences in oral esketamine bioavailability as a result of, for example, genetic differences in cytochrome P450 (CYP)-enzyme activity, liver dysfunction, or concurrent medications (Fanta et al., 2015; Peltoniemi, 2012), and a potential ceiling effect at higher doses. Future studies with larger sample sizes may be needed to better understand dose-response relationships. More important, however, would be the investigation of esketamine plasma concentration and clinical outcomes to optimize dosing regimens.

## Conclusion

This open-label study investigated the real-world effectiveness of repeated oral esketamine in patients with TRD. The study included patients with a wide variety of comorbidities and with levels of treatment resistance that are substantially higher than in other studies on the efficacy of (es)ketamine for depression. After 6 weeks of treatment, beneficial effects were a reason to continue with maintenance treatment in 45.9% of the patients. We observed a significant antidepressant effect and a good safety and tolerability profile, in agreement with other studies investigating oral (es)ketamine for depression. Repeated oral esketamine treatment appears to be a patient-friendly and clinically scalable alternative to other routes of administration. However, long-term studies should investigate the sustainability of the antidepressant effect and possible longer-term adverse effects. In addition, larger RCTs comparing repeated oral esketamine with currently registered treatment options for TRD are warranted.

## Supplemental Material

sj-docx-1-jop-10.1177_02698811251332831 – Supplemental material for Oral esketamine for patients with severe treatment-resistant depression: Effectiveness, safety, and tolerability of a six-week open-label treatment program Supplemental material, sj-docx-1-jop-10.1177_02698811251332831 for Oral esketamine for patients with severe treatment-resistant depression: Effectiveness, safety, and tolerability of a six-week open-label treatment program by Jolien KE Veraart, Sanne Y Smith-Apeldoorn, Annemarie van der Meij, Jan Spijker, Robert A Schoevers and Jeanine Kamphuis in Journal of Psychopharmacology

sj-docx-2-jop-10.1177_02698811251332831 – Supplemental material for Oral esketamine for patients with severe treatment-resistant depression: Effectiveness, safety, and tolerability of a six-week open-label treatment program Supplemental material, sj-docx-2-jop-10.1177_02698811251332831 for Oral esketamine for patients with severe treatment-resistant depression: Effectiveness, safety, and tolerability of a six-week open-label treatment program by Jolien KE Veraart, Sanne Y Smith-Apeldoorn, Annemarie van der Meij, Jan Spijker, Robert A Schoevers and Jeanine Kamphuis in Journal of Psychopharmacology
