# Supplementary material for: Oral esketamine for patients with severe treatment-resistant depression: Effectiveness, safety, and tolerability of a six-week open-label treatment program
Source: J Psychopharmacol. 2025 Apr 25;39(6):559–70. doi: 10.1177/02698811251332831 (PMC12205164; doi:10.1177/02698811251332831)
Supplement: sj-docx-1-jop-10.1177_02698811251332831 – Supplemental material for Oral esketamine for patients with severe treatment-resistant depression: Effectiveness, safety, and tolerability of a six-week open-label treatment program [file sj-docx-1-jop-10.1177_02698811251332831.docx]

**Supplementary Information 1**

**DM-TRD N = 156**

| **Item and specification** | **N** | **%** |
| --- | --- | --- |
| Episode duration |  |  |
| Acute | 12 | 7.7 |
| Sub-acute | 25 | 16.1 |
| Chronic | 118 | 76.1 |
| Symptom severity |  |  |
| Subsyndromal | 0 | 0 |
| Mild | 2 | 1.3 |
| Moderate | 31 | 19.9 |
| Severe without psychosis | 110 | 70.5 |
| Severe with psychosis | 13 | 8.3 |
| Functional impairment |  |  |
| No impairment (GAF 90 - 100) | 0 | 0 |
| Mild impairment (GAF 60 - 90) | 8 | 5.2 |
| Moderate impairment (GAF 30 - 60) | 116 | 75.3 |
| Severe impairment (GAF < 30) | 30 | 19.5 |
| Comorbid anxiety symptoms |  |  |
| Not present | 62 | 40.5 |
| Present, but not fulfilling DSM-IV criteria | 32 | 20.9 |
| Fulfilling criteria ≥ 1 DSM-IV anxiety disorder | 59 | 38.6 |
| Comorbid personality disorder |  |  |
| Not present | 70 | 45.2 |
| Present; not based on formal interview | 38 | 24.5 |
| Present; based on formal interview | 47 | 30.3 |
| Psychosocial stressors |  |  |
| No psychosocial stressor | 35 | 22.4 |
| ≥1 Psychosocial stressor | 121 | 77.6 |
| **Treatment failures in the current depressive episode** |  |  |
| Antidepressants |  |  |
| Level 0: not used | 0 | 0 |
| Level 1: 1 – 2 medications | 25 | 16.1 |
| Level 2: 3 – 4 medications | 49 | 31.6 |
| Level 3: 5 – 6 medications | 41 | 26.5 |
| Level 4: 7 – 10 medications | 27 | 17.4 |
| Level 5: > 10 medications | 13 | 8.4 |
| Augmentation/combination |  |  |
| Level 0: not used | 16 | 10.5 |
| Level 1: 1 – 2 medications | 94 | 61.8 |
| Level 2: 3 – 4 medications | 36 | 23.7 |
| Level 3: 5 – 6 medications | 6 | 3.9 |
| Electroconvulsive therapy |  |  |
| Not used | 61 | 43.6 |
| Used | 79 | 56.4 |
| Psychotherapy |  |  |
| Not used | 10 | 6.5 |
| Supportive therapy | 31 | 20.1 |
| 1 empirically supported psychotherapy | 47 | 30.5 |
| ≥ 2 empirically supported psychotherapies | 66 | 42.9 |
| Intensified treatment |  |  |
| Not used | 33 | 21.0 |
| Day patient treatment | 22 | 14.0 |
| Inpatient treatment | 102 | 65.0 |
