# Supplementary material for: Oral esketamine for patients with severe treatment-resistant depression: Effectiveness, safety, and tolerability of a six-week open-label treatment program
Source: J Psychopharmacol. 2025 Apr 25;39(6):559–70. doi: 10.1177/02698811251332831 (PMC12205164; doi:10.1177/02698811251332831)
Supplement: sj-docx-2-jop-10.1177_02698811251332831 – Supplemental material for Oral esketamine for patients with severe treatment-resistant depression: Effectiveness, safety, and tolerability of a six-week open-label treatment program [file sj-docx-2-jop-10.1177_02698811251332831.docx]

**Supplementary Information 2**

**SAFTEE**

| **Item** | **Moderate discomfort** | | **Severe discomfort** | |
| --- | --- | --- | --- | --- |
|  | N | % | N | % |
| **38. Delayed or absent orgasm** | 1 | 0.8 | 8 | 6.6 |
| 31. Constipation | 3 | 2.4 | 2 | 1.6 |
| 37. Problems with sexual arousal | 0 | 0 | 5 | 4.1 |
| 1. Trouble sleeping | 4 | 3.2 | 0 | 0 |
| 44. Weight loss | 4 | 3.2 | 0 | 0 |
| 14. Headache | 3 | 2.4 | 1 | 0.8 |
| 4. Feeling nervous or hyper | 3 | 2.4 | 0 | 0 |
| 18. Dry mouth | 3 | 2.4 | 0 | 0 |
| 20. Muscle cramps or stiffness | 3 | 2.4 | 0 | 0 |
| 34. Frequent need to urinate | 3 | 2.4 | 0 | 0 |
| 54. Strange taste in mouth | 3 | 2.4 | 0 | 0 |
| 46. Diminished mental | 3 | 2.4 | 0 | 0 |
| 55. Unable to sit still | 3 | 2.4 | 0 | 0 |
| 48. Apathy/Emotional Indifference | 2 | 1.6 | 1 | 0.8 |
| 22. Trouble sitting still | 2 | 1.6 | 0 | 0 |
| 30. Stomach or abdominal discomfort | 2 | 1.6 | 0 | 0 |
| 41. Appetite decreased | 2 | 1.6 | 0 | 0 |
| 11. Abnormal sensations | 2 | 1.6 | 0 | 0 |
| 17. Stuffy nose | 2 | 1.6 | 0 | 0 |
| 43. Weight gain | 2 | 1.6 | 0 | 0 |
| 49. Dizziness when you stand up | 2 | 1.6 | 0 | 0 |
| 7. Poor memory | 1 | 0.8 | 1 | 0.8 |
| 53. Clenching of teeth at night | 1 | 0.8 | 1 | 0.8 |
| 52. Hot flashes | 1 | 0.8 | 1 | 0.8 |
| 36. Loss of sexual interest | 0 | 0 | 2 | 1.6 |
| 13. Dizziness or faintness | 1 | 0.8 | 0 | 0 |
| 16. Ringing in ears or trouble hearing | 1 | 0.8 | 0 | 0 |
| 3. Feeling drowsy or sleepy | 1 | 0.8 | 0 | 0 |
| 2. Nightmares or other sleep disturbance | 1 | 0.8 | 0 | 0 |
| 5. Weakness or fatigue | 1 | 0.8 | 0 | 0 |
| 9. Feeling strange or unreal | 1 | 0.8 | 0 | 0 |
| 10. Hearing or seeing things | 1 | 0.8 | 0 | 0 |
| 12. Numbness or tingling | 1 | 0.8 | 0 | 0 |
| 19. Drooling or increased salivation | 1 | 0.8 | 0 | 0 |
| 21. Muscle twitching or movements | 1 | 0.8 | 0 | 0 |
| 26. Heartbeat rapid or pounding | 1 | 0.8 | 0 | 0 |
| 27. Trouble catching breath or hyperventilation | 1 | 0.8 | 0 | 0 |
| 33. Difficulty starting urination | 1 | 0.8 | 0 | 0 |
| 47. Difficulties finding words | 1 | 0.8 | 0 | 0 |
| 51. Hair thinning/loss | 1 | 0.8 | 0 | 0 |
| 15. Blurred vision | 0 | 0 | 1 | 0.8 |
| 35. Menstrual irregularities | 0 | 0 | 1 | 0.8 |
| 6. Irritable | 0 | 0 | 0 | 0 |
| 8. Trouble concentrating | 0 | 0 | 0 | 0 |
| 23. Tremor or shakiness | 0 | 0 | 0 | 0 |
| 24. Poor coordination or unsteadiness | 0 | 0 | 0 | 0 |
| 25. Slurred speech | 0 | 0 | 0 | 0 |
| 28. Chest pain | 0 | 0 | 0 | 0 |
| 29. Nausea or vomiting | 0 | 0 | 0 | 0 |
| 32. Diarrhea | 0 | 0 | 0 | 0 |
| 39. Sweating excessively | 0 | 0 | 0 | 0 |
| 40. Fluid retention or swelling | 0 | 0 | 0 | 0 |
| 42. Appetite increased | 0 | 0 | 0 | 0 |
| 45. Skin rash or allergy | 0 | 0 | 0 | 0 |
| 50. Bruising | 0 | 0 | 0 | 0 |
